# Lipidic Liquid Crystalline Cubic Phases and Magnetocubosomes as Methotrexate Carriers

**DOI:** 10.3390/nano9040636

**Published:** 2019-04-19

**Authors:** Monika Mierzwa, Adrianna Cytryniak, Paweł Krysiński, Renata Bilewicz

**Affiliations:** Faculty of Chemistry, University of Warsaw, Pasteura 1, PL 02-093 Warsaw, Poland; monikaszlezak89@wp.pl (M.M.); a.cytryniak@gmail.com (A.C.); pakrys@chem.uw.edu.pl (P.K.)

**Keywords:** methotrexate, cubic phase, magnetocubosomes, monoolein, liquid crystalline phase, drug delivery system, alternating magnetic field

## Abstract

The release profiles of methotrexate, an anticancer drug, from the monoolein liquid crystalline cubic phases were studied. The cubic phases were used either in the form of a lipidic film deposited onto a glassy carbon electrode surface or in the dispersed form of magnetocubosomes, which are considered a prospective hybrid drug delivery system. Commonly, cubosomes or liposomes are employed, but not in the case of toxic methotrexate, known to block the receptors responsible for folate transport into the cells. The release profiles of the drug from the lipidic films were monitored electrochemically and described using the Higuchi model. They were also modified via changes in temperature; the release was faster, although it deviated from the model when the temperature was increased. Cubic phase nanoparticles (magnetocubosomes) containing hydrophobic magnetic nanoparticles placed in an alternating magnetic field of low frequency and amplitude, stimulated drug release from the suspension, which was monitored spectroscopically. These new biocompatible hybrid nanomaterials in the dispersed form allow to control the release of the drug at the appropriate sites, can be easily separated or relocated under external magnetic field and await further investigations of their in vitro cytotoxicity and in vivo biodistribution.

## 1. Introduction

Methotrexate (MTX), also known as amethopterin, is an antimetabolite of folic acid. Its molecular structure differs from that of the folic acid (FA) in two ways. First, MTX has a four-amino group instead of a hydroxyl group on the pteridine ring, and second, it has a methyl group at the N^10^ position (Scheme S1).

MTX blocks folate synthesis by inhibiting enzyme dihydrofolate reductase, which synthesizes the active form of folate required for DNA and protein synthesis, leading to a major decrease in cancer cell proliferation [1]. It is widely used for the treatment of acute leukemia, malignant lymphoma, osteogenic sarcoma, choriocarcinoma, and other cancers (breast, head, neck, pulmonary, and epidermoid). MTX is also used in the treatment of rheumatoid arthritis or other autoimmune diseases [2,3]. MTX is currently one of the most studied antitumor chemotherapeutic drugs since its therapeutic utility is limited by its high toxicity in the healthy cells of patients whose levels of folates are also seriously reduced [4]. The principal side effects of MTX treatment are myelosuppression, thrombocytopenia, anemia, gastrointestinal mucositis, hepatitis, nausea, fatigue, headache, and/or dyspnea [3,4,5,6].

In light of the systemic side effects of anticancer drugs, the design of drug carriers is very important. There are several methods for limiting MTX toxicity through its administration by means of carriers or targeting carriers, e.g., liposomes [7,8], silica materials [9,10], lipid nanoparticles [11,12], chitosan nanoparticles [13], polymeric micelles with magnetic nanoparticles [14], nanorods [15], implants [16], and nanogels [17,18].

A promising carrier is the liquid-crystalline lipidic cubic phase (LCP), which possesses a variety of desirable properties making it a good candidate for drug delivery systems (DDS) [19]. The advantages of nonlamellar LCP nanoparticles such as cubosomes over planar structures and liposomes are connected with the larger membrane surface area to nanoparticle volume and increased drug-loading capacity thanks to the huge internal lipid‒water interface. The curvature of the bilayer can be tuned independently of the nanoparticle size, channel size, and the thickness of the lipid layer can be controlled by the selection of lipids used for the formation of the cubic phase and application of dopants [20,21]. An outer block co-polymer stabilizes and makes targeting the carrier possible. Cubosomes retain the internal structure of parent bulk phase, however, in the dispersed system the viscosity of the bulk phase is reduced, which makes it easier to apply.

LCP composed of monoolein (MO) is non-toxic, bioadhesive, and biodegradable. The biodegradability of LCP is due to the fact that monoolein undergoes slow lipolysis in contact with the esterase found in the tissues. The highly structured inverse bicontinuous LCP is composed of two identical, nonintersecting aqueous channels that are surrounded by curved lipid bilayers and can accommodate large amounts of active molecules of any polarity [22]. LCPs are stable in the presence of water, which is an important property in light of their perspective applications for DDS. The rate of drug release from liquid crystals can be modified by either changing the symmetry of the liquid crystal, the dimension of the water channel, or the pH of the environment. Generally, this can be achieved through the manipulation of the lipid composition [23,24,25,26]. Previously, we studied the physical and chemical properties of hybrid materials composed of LCPs bearing hydrophilic and hydrophobic magnetic nanoparticles as a material for drug delivery where the outcome was promising [27]. Magnetic nanoparticles and ions are currently of great interest in research related to medical applications [28] and are used as contrast agents for theranostics in medical imaging applications [29]. Few reported studies have examined the effect of the addition of nanoparticles to the liquid crystalline hexagonal phase [30], liposomes [31,32], and cubosomes [33,34,35], creating a magnetic responsive system. Magnetic nanoparticles with hydrophobic organic shell, localized within the bilayer lipid membrane of liposomes were reported to mechanically rupture these structures in low-frequency alternating magnetic field (LF-AMF) of 50 Hz and 10 mT field strength. No magnetic hyperthermia was involved in such field. When the vesicles were loaded with doxorubicin—A potent cytostatic drug, the relatively mild magnetic stimulus triggered the release of the drug [32]. Montis et al. studied in situ the diffusion of two fluorescent tags, the hydrophobic Octadecyl Rhodamine B and the hydrophilic Rhodamine 110, from the magnetocubosomes using Fluorescence Correlation Spectroscopy during the application of LF-AMF. The application of LF-AMF accelerated the release of the hydrophilic molecules from the water channels of magnetocubosomes [33].

It is known that the use of external alternating magnetic field may adversely affect tissues, including normal tissue toxicity. This effect can be considered in terms of heat production by the interaction of AMF with tissue by its direct heating, induction heating of a material embedded within the tissue (here—magnetic nanoparticles) or both. The second type of heat production is localized within the direct vicinity of superparamagnetic iron oxide nanoparticles and is considered for, e.g., cancer treatment, via the classical hyperthermia response. For the hyperthermia-related treatment the AMF frequencies of kHz to MHz are used, with magnetic field amplitude of ca. 40 to 100 mT [36]. In our studies low-frequency AMF (house frequency) of 50 Hz and 10 mT amplitude is used, and in fact, hyperthermia effect can be neglected as discussed below in Section 3.3. On the other hand, interaction of AMF with tissue can produce heat directly, even though this “material” is of very low conductivity as compared with typical conductive material, like metals. The mechanism that dominates this nonspecific heating results from the production of eddy currents (Foucault’s currents) producing heat that scales as: SAR_EC_ ~ f^2^ H^2^ r^2^, in which SAR_EC_ is the tissue-specific absorption rate [W/g of tissue], r is the radius of affected region, f and H are the AMF frequency and amplitude, respectively. It was reported [36] that no adverse effects were observed for BALB/c athymic nude mice for AMF amplitudes below 70 mT even at continuous power application (100% duty) for up to 20 min. Generally, it is accepted that high-amplitude AMF (<130 mT) and a frequency of ca. 150 kHz was well tolerated, provided fast heat dissipation. Again, we stress that in the studies reported in this work we have used 50 Hz (3000 times lower frequency) and 10 mT field (4-fold smaller), therefore, it will not induce nonspecific tissue heating.

In this study, we focus on the delivery of MTX loaded monoolein hybrid systems. The MTX load and release studies were conducted for the monoolein cubic phase and cubosomes with and without hydrophobic magnetic nanoparticles present in the lipidic part of the carrier. By taking advantage of the presence of hydrophobic magnetic nanoparticles localized in the lipidic part of magnetocubosomes, we used an alternating magnetic field (AMF) with low frequency to stimulate the release of the drug from the carriers.

## 2. Experimental

### 2.1. Materials

All chemicals were of the highest quality and commercially available. Monoolein (1-oleoyl-rac-glycerol) (MO) (˃99% pure) was purchased from Hampton Research (Aliso Viejo, CA, USA) and used as received. Methotrexate (N-[4{[2,4-diamino-6-pteridinyl-methyl]-methylamino}benzoyl] glutamic acid) (MTX) was obtained from Fluka (˃98% pure, Munich, Germany) and used without further purification. Pluronic F-127 was purchased from Sigma-Aldrich (St. Louis, MO, USA). A 0.2 M phosphate buffer was prepared by titrating 0.2 M di-sodium hydrogen phosphate (˃99% pure, ChemPur, Karlsruhe, Germany) with 0.2 M sodium di-hydrogen phosphate (˃99% pure, Polish Chemicals Co., Poland) to pH 7.4. All solutions were prepared using Milli Q water (18.2 MΩ·cm^−1^; Millipore, Bedford, MA, USA). To synthesize the hydrophobic nanoparticles, FeCl_3_•6H_2_O (≥98% pure, Sigma-Aldrich), FeCl_2_•4H_2_O (≥99% pure, Sigma-Aldrich), NH_4_OH (25% aqueous solution in H_2_O, Chempur), and oleic acid (>99% pure, Sigma-Aldrich) were used.

### 2.2. Preparation of Cubic Phases and Cubosomes

The LCPs were prepared by adding the appropriate amount of MTX solution to a 0.1 M phosphate buffer in a glass vial with molten MO to obtain 59/1/40, 59.5/0.5/40, or 59.75/0.25/40% (w/w) for the phase MO/MTX/buffer.

Magnetic nanoparticles (MNPs) used to prepare hybrid cubic phases were synthesized as described in our previous report [27]. Briefly, we used the co-precipitation method with oleic acid as the capping agent. The synthesis was carried out under vigorous stirring and an argon atmosphere. As the first step, FeCl_2_•4H_2_O (0.023 mol) and FeCl_3_•6H_2_O (0.046 mol) were dissolved in 150 mL of deionized water. The mixture was heated to 45 °C and then 11 mL of 25% ammonia was added, which caused the appearance of a black precipitate of nanoparticles. After half an hour, 3 mL of oleic acid was added. Then, the suspension was heated to 80 °C and kept at this temperature for one hour. Afterwards, the Fe_3_O_4_ nanoparticles coated with oleic acid were washed several times with deionized water and ethanol under magnet-assisted sedimentation to remove the excess oleic acid. After thorough washing, the nanoparticles of ca. 7 ± 2 nm in diameter were transferred to hexane as a stable suspension of controlled density. The size distribution and mass contribution of the organic shell (ca. 35%) were discussed in our previous work [27,32]. The magnetic characterization of dry powder nanoparticles was carried out with a QD vibrating sample magnetometer VSM, working at 300 K and stabilized to 0.01 K accuracy, over the magnetic field range from 2.0 to +2.0 T. The saturation magnetization of the as synthesized, oleic acid-coated nanoparticles was ca. 43 emu/g, which was comparable with the literature and our previous work [32].

To prepare the hybrid LCPs, the appropriate amount of MNP dispersion in hexane was added to the molten MO and sonicated. The mixture was left in a desiccator to evaporate the solvent. Then, MTX, in a phosphate buffer solution was added to the mixture. The ratio of the components of the hybrid system MO/NPs/MTX/buffer was 57.5/2/0.5/40% (w/w). The samples were left in tightly sealed vials at room temperature in the dark until they became homogenous and transparent.

The procedure of preparing the magnetocubosome dispersion started with mixing molten MO and MNP dispersion in hexane (34.5 mg of MNPs per 1 g of MO). After the evaporation of the solvent, MTX was added (8.7 mg MTX per 1 g of MO). The solution was then mixed and followed by sonication until it became homogeneous. Then, the 1% F-127 solution was added to obtain 94% w/w and the mixture was dispersed by ultrasonication using a Sonics Vibra cell for 20 min (3 s “on”, 5 s “off”) at an amplitude of 40% of maximum output (130 watt, 30 kHz).

#### 2.2.1. Dynamic Light Scattering (DLS) and Zeta Potential

The hydrodynamic diameter of the cubosomes was determined using a Malvern Zetasizer instrument (Nano ZS, Malvern, UK) fitted with a 4 mW He−Ne laser (λ = 632.8 nm) as the light source with a scattering angle of 173°. Samples were equilibrated for 2 min before measurement. The zeta potential was measured using the same instrument. The values were reported as averages from 3 measurements of each sample.

#### 2.2.2. Cryogenic Transmission Electron Microscopy (Cryo-TEM)

A droplet of the cubosome sample was deposited on a lacey copper grid. The sample was then blotted with filter paper inside a climate chamber (Vitrobot Mark IV, FEI, Hillsboro, OR, USA). The temperature was kept at 22 °C and 100% humidity. Afterwards, the grid was plunged into a liquid ethane bath cooled with liquid nitrogen. The cooled gird was maintained at a temperature of −170 °C using a cryo holder 626 (Gatan Inc., Pleasanton, CA, USA). Specimens were observed with a TECNAI transmission electron microscope (Hillsboro, OR, USA) operating at 120–200 kV acceleration voltage equipped with a 4 k CCD camera FEI Eagle (Hillsboro, OR, USA).

### 2.3. Electrochemical Measurements

Electrochemical measurements were performed using a CH Instruments bipotentiostat model CHI750B (Austin, USA). The three-electrode system consisted of a glassy carbon electrode (GC macroelectrode A = 0.07 cm^2^, GC microelectrode A = 98.4 μm^2^), Ag, AgCl|3 M KCl_aq_ reference electrode, and Pt foil as a counter electrode. The GC electrodes were polished each time before measurement with alumina powder (1.0, 0.3, and 0.05 μm) on a polishing cloth. The electrodes were rinsed with ultrapure water, sonicated in an ultrasonic bath and left to dry in air. Then, the working space of the GCE was covered with a ca. 1 mm thin film of the cubic phase (which corresponded to ca. 5 mg of the phase). The measurements were performed at 25 °C and 37 °C using a BVT MT-1 minithermostat to maintain constant temperature. The electrochemical measurements were performed in a glass cell (BVT company, Brno, Czech Republic) with a volume of 10 mL. The solution in the cell was deoxygenated by passing high purity argon through the solution for 15 min before the measurement and the flow of pure gas over the solution was maintained during the experiment. We performed cyclic (CV), differential pulse (DPV) or square wave (SWV) voltammetry experiments on GC electrodes modified with a cubic phase. The CV experiments were carried out at various scan rates within the constant potential window of −0.2 V (starting point) to −1.2 V. For each type of cubic phase, the experiments were done in triplicate. The release profiles of MTX from the LCPs were obtained by recording the DPV peaks of MTX reduction. The pulse amplitude was 50 mV, pulse width 70 mV, and a pulse period 0.2 s. The saturation of the empty cubic phase with MTX was monitored also in the SWV mode with the following parameters: frequency 25 Hz, potential increment 2 mV, and amplitude 0.01 V.

### 2.4. Modeling of the Kinetics of Drug Release

There are several literature models that describe the drug release from different matrices [37]. In accordance with our previous work [27], to determine the kinetics of the drug elution from the LCPs, the Korsmeyer–Peppas model was chosen as it describes the controlled drug release from polymeric systems similar to cubic phases. The kinetics of the drug release, according to this model, are described by the following equation:

(1) $$\frac{M_{t}}{M_{\infty }}={\mathrm{kt}}^{n},$$

where M_t_/M_∞_ is the fraction of drug released at time t versus t_∞_; *k* is the release rate constant; and the *n* value gives details on the release mechanism of the drug. To determine the value of *n*, the logarithm of less than the initial 60% of the release data values were fitted versus the log time. In the case of a cylindrical surface of a drug-containing carrier, when *n* = 0.45, the diffusion mechanism corresponds to Fick’s law; for *n* between 0.45 and 0.89, the transport is non-Fickian; *n* = 0.89 corresponds to Case-II transport; and *n* > 0.89 for Super Case-II transport [37].

When *n* ≈ 0.5, the Korsmeyer–Peppas approach is simplified to the Higuchi model, which describes the drug release from semi-solid matrices. The Higuchi model is described by the following expression:

M_t_/M_∞_ = k_H_√t, (2)

where k_H_ is the Higuchi constant. 

The drug release profiles from the cubic mesophase films were evaluated from measurements performed using both spectrophotometry and voltammetry. The former reflects the increasing concentration of the drug in the solution while electrochemistry shows the removal from the films present at the electrode monitored by the underlying electrode. The spectrophotometry approach is more common, however, the electrochemical approach shows the release from the film and any solution phenomena such as the adsorption of the released drug on glassware will not affect the result. In the case of magnetocubosomes, only UV-Vis spectrophotometry was employed.

### 2.5. Magnetic Field Generator

The magnetic field generator was a custom-made inductor coil connected to an alternating electric power outlet (20 V, 50 Hz). The void of the inductor (stator) coil provided space for a quartz cuvette with a suspension of magnetocubosomes. Due to the design of the set-up, the magnetic field inside the inductor and quartz cuvette was not isotropic and its maximum value was measured as 10 mT. A constant temperature was maintained (25 °C) during the release experiments and the coil was cooled using a fan [32].

### 2.6. Spectroscopic Measurements

The UV-Vis measurements were also used to drug elution studies. The GCE was modified with a thin film of cubic phase containing 1.00% MTX and was placed in the cell filled with 10 mL of 0.1 M phosphate buffer at 25 °C. The cell was placed on a magnetic stirrer with gently stirring to keep the same concentration of drug in the entire volume of the cell. After a specified time, 2 mL of the solution was transferred to an acrylic cuvette and placed in the spectrophotometer. After the measurement the solution was returned to the cell. Experiments showing AMF-stimulated drug release from hybrid materials were performed using a UV-Vis Cary 60 spectrophotometer (Agilent Technologies, Warsaw Poland) with a 1 cm quartz cuvette in the wavelength range from 600 to 250 nm. A series of samples, each containing 0.5 mg/mL magnetocubosome dispersion in 0.1 M phosphate buffer at pH 7.4, was prepared. Each of the samples was subsequently placed in the cuvette inside the inductor coil for a specified time. Then, the cuvette was removed from the inductor coil and the absorbance was measured at 303 nm. For each experiment, a fresh sample was used.

The spectroscopic measurements were also used to calculate the entrapment efficiency (%) of MTX in magnetocubosomes. The freshly prepared magnetocubosomes were diluted ten times. Then, the diluted sample was placed in an Amicon Ultra-0.5 centrifugal filter unit with an Ultracel-3 membrane (NMWL 3 kDa, Sigma Aldrich, Munich, Germany) and centrifuged for 20 min at 6000 rpm to separate the unbound drug. The separated aqueous phase with free drug was ten times diluted in 0.1 M phosphate buffer and the absorbance was measured at 303 nm. The entrapment efficiency (EE) was determined using the following expression:

(3) $$EE=\;\frac{C_{T}-C_{F}}{C_{T}}\cdot 100\%,$$

where *C*_T_ is the concentration of the drug added to the cubosome during preparation and *C*_F_ is the concentration of the free drug in the ultrafiltrate detected after centrifugation.

The standard calibration curve for MTX solution in 0.1 M phosphate buffer at pH 7.4 was developed from at least nine dilutions in the concentration range of 20.0–0.89 μg/mL. The absorption spectra of the MTX buffer solutions were measured in the wavelength range of 600–250 nm with the characteristic λ at 303 nm. All measurements were performed at 25 °C.

## 3. Results and Discussion

### 3.1. Structural Characterization of the MTX-Doped Cubic Phases 

**Figure 1:**
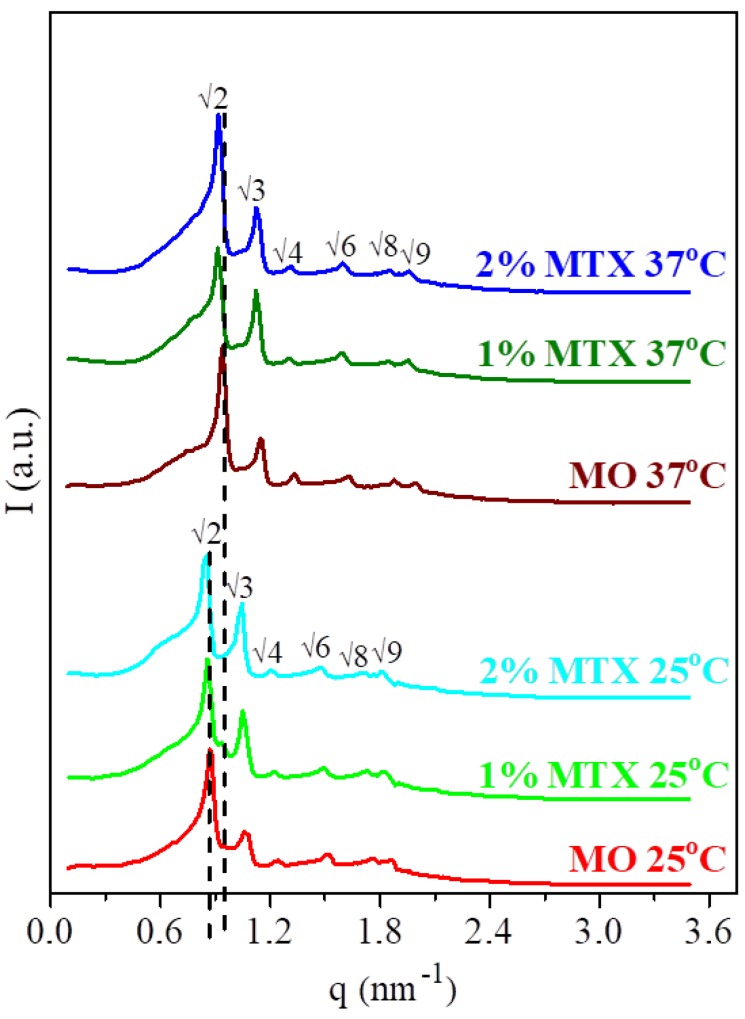
Comparison of SAXS spectra for the bulk cubic phases with 40% w/w of buffer and different amounts of MTX at 25 °C and 37 °C. Peaks are indexed for a double diamond cubic phase. The dashed lines show the position of the first peak for MO phase at 25 °C and 37 °C.

It is well known that a monoolein/water mixture in a ratio of 60/40% w/w forms a bicontinuous cubic phase characterized by *Pn3̅m* symmetry [38,39,40]. The presence of MTX up to 2% w/w did not compromise the internal structure of the monoolein bicontinuous cubic phase. The size of the drug was smaller than the length of the unit cell of the cubic phase. The SAXS data of the pure MO phase and those doped with 2% and 1% w/w of MTX at 25 °C and 37 °C are shown in Figure 1. The peak positions of a cubic lattice were observed at both temperatures. These peak positions were consistent with the cubic phase of the *Pn3̅m* space group (characteristic Bragg diffractions: √2:√3:√4√6:√8:√9 are shown near the corresponding peaks in Figure 1). The calculated values of the lattice parameter, lipid length, and diameter of the water channels are listed in Table 1. The values were calculated from the SAXS spectra (for more details see S2 and Kulkarni et al. [22]). Incorporation of MTX into the monoolein phase did not affect the lattice parameter or the size of the water channels (Table 1), and noticeable differences of ca. 0.1 nm were on the verge of error.

### 3.2. Electrochemical Measurements

#### 3.2.1. Methotrexate Incorporated in the Monoolein Cubic Phase

MTX is an electroactive molecule that undergoes two redox processes: (1) The oxidation of MTX pyrazine moiety to 7-hydroxymethotrexate, which is an irreversible process occurring at positive potentials with the exchange of 2e^−^/2H^+^; and (2) The reduction at negative potentials of the diamino-pteridinyl moiety to its 5,8-dihydro derivative via transfer of 2e^−^/2H^+^, which is also reversible (Scheme S3) [41].

**Figure 2:**
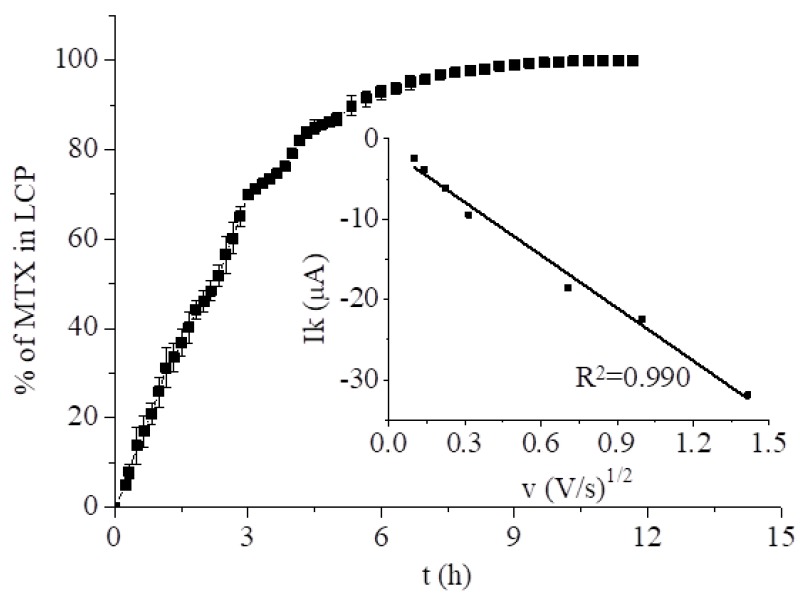
Saturation of LCP with the drug from 2.33 mM MTX solution in 0.2 M phosphate buffer monitored by SWV. Inset: Dependence of the CV reduction peak current at −0.790 V vs. the square root of the scan rate for the cubic phase layer phase with MTX after 12 h of saturation.

The electrochemical behavior of MTX was investigated using cyclic (CV), square wave (SWV), and differential pulse voltammetry (DPV) in 0.2 M phosphate buffer, pH 7.4. Previous reports have shown that the electrochemical response of MTX to CGE differs depending on the buffer pH [41,42]. Even though the obtained peaks were well resolved in acidic media, our main goal was to examine the drug’s behavior at physiological pH. We began our investigations by examining the redox processes of MTX at an electrode covered with LCP saturated with the drug. This was achieved by dipping the electrode modified with monoolein LCP in a 10 mL, 2.33 mM solution of MTX in 0.2 M phosphate buffer at room temperature. The process of cubic phase saturation with MTX was monitored using the highly sensitive SWV method. After 15 min, the peak correlating to drug reduction was observed. Saturation was reached after 8 h (Figure 2). When a constant SWV peak current was attained, cyclic voltammograms were recorded at different scan rates. The dependencies of the reduction peak currents on the square root of the scan rate was linear (*R*^2^ = 0.990), proving the diffusion control of the electrode processes (Figure 2 inset).

**Figure 3:**
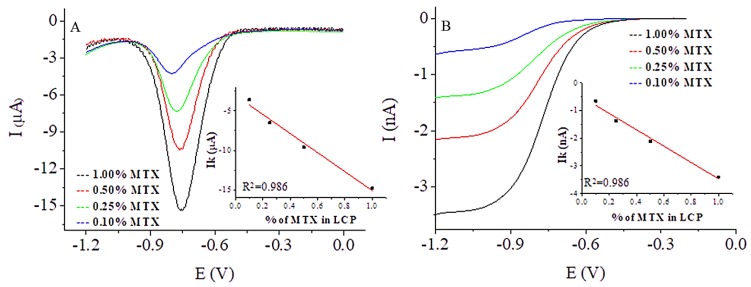
DPV recorded from a GCE (**A**) and CV from a GC microelectrode (**B**) modified with the cubic phase containing 1.00, 0.50, 0.25, and 0.10% w/w of MTX at pH 7.4. Inset: Dependence of the MTX response on the drug concentration in the LCP.

In the next step, MTX was incorporated into the monoolein cubic phase at several concentrations: 0.10, 0.25, 0.50, and 1.00% w/w. Drug-loaded cubic phases were used to modify the normal size GCE and GC microelectrodes. The working space of the electrodes was modified with a thin film of cubic phase (ca. 1 mm) containing MTX and transferred to the cell filled with 10 mL of deoxygenated phosphate buffer at room temperature. The response of the drug in negative potentials was monitored using DPV for the normal sized electrode and CV for the microelectrode. In the case of GCE, the transport to the electrode surface was determined by linear diffusion and recorded as a peak. The DPV reduction peak currents of the drug appeared ca. −0.790 V and increased with MTX concentration (Figure 3A). In the case of the microelectrode, the diffusion of the electroactive molecules was spherical and the CV response was in the form of a wave (Figure 3B). The wave height increased with the amount of MTX in the LCP. In both cases, the dependence of the MTX response on the drug concentration in the LCP was linear (Figure 3 inset).

**Figure 4:**
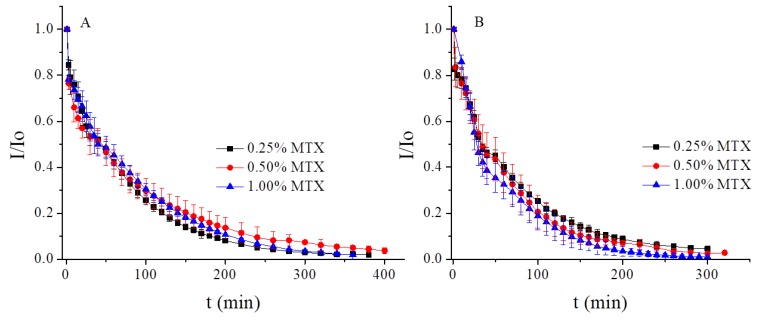
Release profile of MTX from LCPs at pH 7.4 at 25 °C (**A**) and 37 °C (**B**).

Drug elution studies of MTX from monoolein cubic phases were conducted at several concentrations: 0.25, 0.50, and 1.00% w/w. The spectroscopy and voltammetry measurements were performed under an argon atmosphere at 25 °C and 37 °C. The drug release profiles were investigated based on the changes in the DPV voltammograms with time counting from the moment of immersion of the electrodes in the pristine buffer are shown in Figure 4. Over the course of time, the peak current decreased, which directly shows the elution of the drug. The dependence of the peak current on measurement time is shown in Figure S4. The results were plotted as the normalized current (I/Io) vs. time and are presented in Figure 4. Figure 4A demonstrates the elution of the drug at room temperature. The profiles of the drug release were similarly independent of the initial concentration of the drug in the cubic phase. In all cases, the total elution was reached after ca. 350 min, and 50% w/w MTX (T_50_) was released after 42 min for 0.25 and 0.50% w/w MTX and after 40 min for 1.00% w/w MTX. Figure 4B shows the elution of MTX at 37 °C, with the total elution of the drug being achieved after 250 min. T_50_ occurred after ca. 32 min for 0.25 and 0.50% MTX and 29 min for 1.00% MTX. These results can be attributed to the faster moving molecules at higher temperatures.

**Figure 5:**
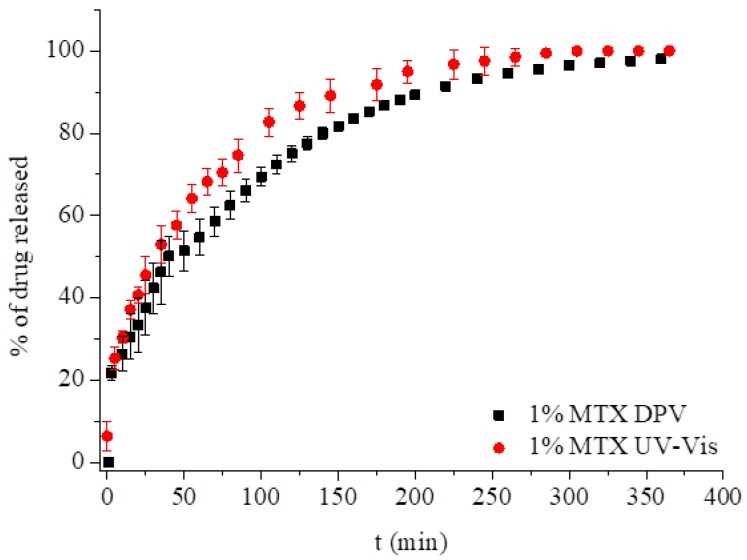
Release profiles of MTX from LCPs measured with UV-Vis spectroscopy and differential pulse voltammetry at pH 7.4 at 25 °C.

For comparison, we measured the elution of 1.00% w/w of MTX from LCP using UV-Vis at 25 °C, keeping the same conditions as in the electrochemical measurements. The drug release profile was investigated based on the increasing concentration of the drug in the solution surrounding the GCE modified with cubic phase. The comparison of release profile of 1% MTX measured with spectroscopy and voltammetry is presented in Figure 5. The profiles of the drug elution were similar for the first 60 min and after 200 min. The total elution was achieved at the same time, but the T_50_ measured with UV-Vis was achieved after ca. 30 min, 10 min earlier than using the electrochemical approach.

All of the drug release data were plotted as a log percentage of the drug release vs. log time [43,44]. The slope of the plot allowed for the evaluation of the exponent *n* in the Korsmeyer–Peppas model. The results obtained for MTX obtained using DPV and UV-Vis approaches at 25 °C gave an *n* value close to 0.45, which means that the diffusion mechanism corresponded to Fick’s laws and the release mechanism proceeded according to the Higuchi model. With increasing temperature, the *n* value in all cases started to deviate from 0.45, indicating the influence of the LCP matrix and a transition to non-Fickian transport (see Table 2).

#### 3.2.2. Behavior of MTX Incorporated into Hybrid LCP Systems

**Figure 6:**
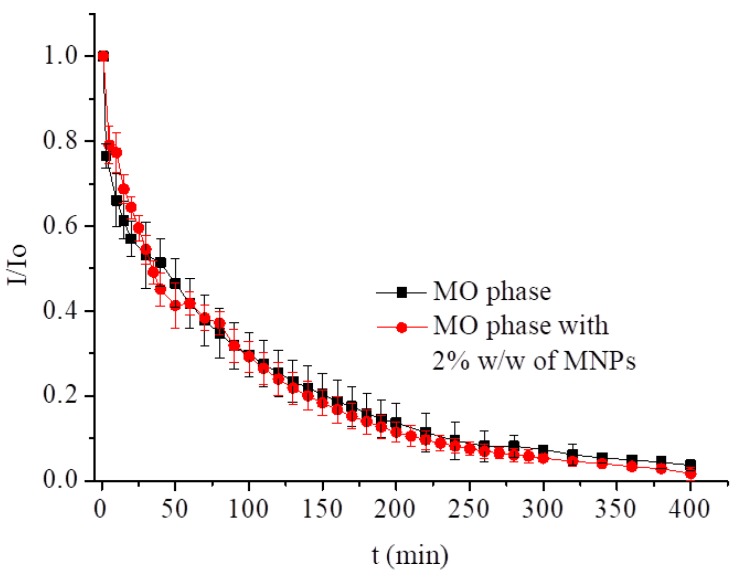
Release profiles of MTX from LCPs with and without magnetic nanoparticles at pH 7.4 at 25 °C.

We have previously reported that the incorporation of up to 2% w/w of hydrophobic magnetic nanoparticles (MNPs) had no effect on the phase symmetry [27]. Due to their size (ca. 7 nm) and lipid coating, MNPs are located mainly in the lipidic domain of the LCP while the water channels remain free. Therefore, the magnetic nanoparticles do not block access of the drug to the electrode surface. A hybrid phase with MTX led to a similar outcome. MTX release profiles from the monoolein hybrid cubic phase containing 2% w/w of MNPs and 0.5% w/w of drug were monitored using the DPV method (Figure 6 and Figure S5). The measurements were carried out at 25 °C under an argon atmosphere. Incorporation of 2% w/w MNPs resulted in an increase of the initial peak current of MTX by 4 μA, without a change in the peak potential, −0.790 V (Figure S5). In the presence of MNPs, the mechanism of the drug release at room temperature was similar to the MO phase. The total elution of MTX was maintained at the same level as the pure monoolein phase. In this case, the value of T_50_ was 35 min and the *n* value of ca. 0.45 suggests that the drug was released according to Fick’s law (see Table 2).

#### 3.2.3. MTX Incorporated into Magnetocubosomes

**Figure 7:**
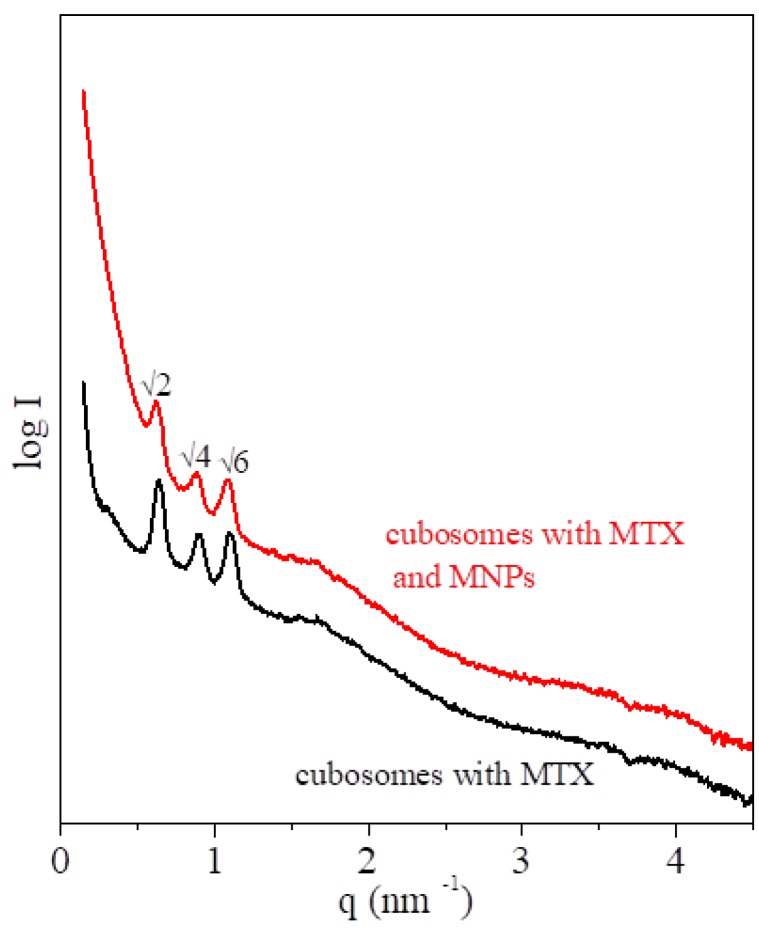
SAXS spectra of the cubosomes and magnetocubosomes with methotrexate at room temperature (25 °C).

Nanoparticles of the hybrid cubic phase, magnetocubosomes, can be used for magnetic drug targeting, hyperthermia treatment, or magnetic resonance imaging [40] due to their small size and magnetic properties that allow them to move in a magnetic field. When loaded with the appropriate anti-cancer drug such as MTX, they can serve as a platform for drug targeting delivery systems. Cubosomes and magnetocubosomes loaded with MTX were prepared, and their characteristics evaluated. The cubic nature of the LCP nanoparticles was characterized by SAXS. The SAXS pattern for cubosomes with MTX showed the relative positions of the diffraction signals—√2:√4:√6—corresponding to the *Im3̅m* space group (Figure 7, black line). In the case of the magnetocubosomes, identical signals were observed (Figure 7, red line). The crystallographic lattice parameter was 13.9 nm and 14.3 nm for the cubosomes and magnetocubosomes, respectively. The diameter of the magnetocubosomes doped with MTX was evaluated using DLS measurements, which was ca. 130 nm, and the zeta potential was ca. –30 mV (Figure S6). The entrapment efficiency of MTX in the magnetocubosomes was evaluated using Equation (3) and a standard calibration curve for MTX solution in 0.1 M phosphate buffer at pH 7.4 (Figure S7). The load efficiency was 63.1%.

Cryo-TEM allows for the imaging of the cubosomes without any changes of their structure. The Cryo-TEM images for cubosomes/magnetocubosomes with MTX showed a well-ordered structure inside the particles (Figure S8). This highly ordered internal structure of the magnetocubosome provides an extensive hydrophobic–hydrophilic interfacial area, e.g., for the solubilization of different drugs and offers a convenient way of delivering the drug into the body. Additionally, the MNPs were visible inside the magnetocubosomes; hence the magnetocubosomes were expected to be sensitive towards magnetic fields. In the Supplementary Video S9 a link to a film showing the movement of magnetocubosomes in the field of the magnet can be found.)

### 3.3. Low-Frequency Alternating Magnetic Field (AMF)-Stimulated Drug Release

The effect that magnetic nanoparticles have on drug release as well as other molecules from various lipidic structures under the influence of the external magnetic field has been well documented. Vallooran et al. [30] showed that under a constant magnetic stimulus, the hexagonal phase with MNPs aligns the orientation of the hexagonal domains in the direction of the external (constant) magnetic field, thus facilitating the transport of a hydrophilic drug across the liquid crystalline phase in this direction. Two cases were considered: when the magnetically induced orientation of the hexagonal domains in the membrane was in the direction of diffusion, it facilitated transport of a hydrophilic drug, and when the forced position of the hexagonal domains was perpendicular to the diffusion direction, it resulted in slower drug diffusion. Application of LF-AMF increased the release of the hydrophilic molecules from the water channels of magnetocubosomes [33]. Mendozza et al. showed that the monoolein cubic phase with MNPs subjected to AMF for 10 min undergoes transition from *Pn3̅m* to H_II_ [45].

**Figure 8:**
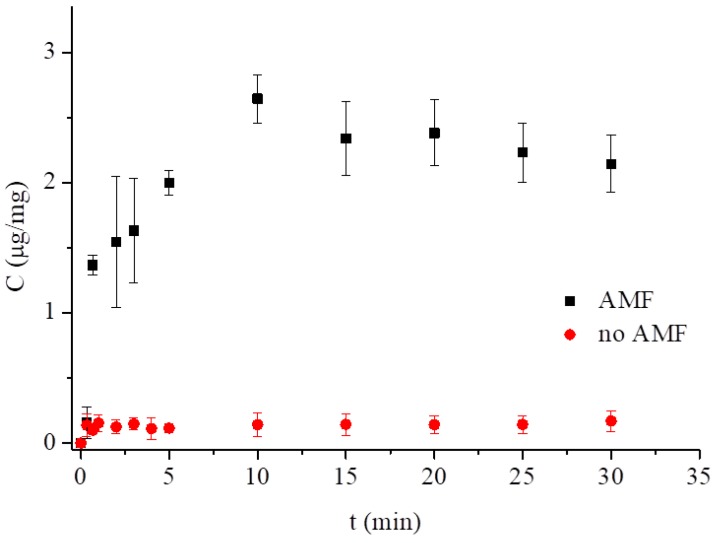
MTX released from magnetocubosomes vs. time, spontaneously and caused by LF-AMF at pH 7.4 and 25 °C.

Other reports [31,33,35,46,47] relied on the so-called magnetothermal effect observed in an alternating magnetic field. This effect reveals itself as temperature rises, transferring heat to the immediate vicinity when the magnetic nanoparticles are exposed to the alternating magnetic field of sufficiently high frequency (typically from several kHz to hundreds of kHz) due to the Néel and Brownian relaxation [48]. This heat transfer affects the physicochemical properties of the lipidic structures, triggering drug release from lipid mesophases [30,33]. Since the heating efficiency, a prerequisite for the magnetothermal effect, varies as ω^2^τ/(1 + ω^2^τ^2^), where ω is the magnetic field frequency and τ is the effective relaxation time, under the LF-AMF conditions used in this work (50 Hz AMF, 10 mT, 20 V), the relaxation time was only ca. 10^−6^ s [49], therefore the magnetothermal effect as the main stimulus for drug release has to be excluded. Another mechanism of triggered drug release includes changes of the nanomechanical properties of the hybrid MNP–lipidic material. The incorporation of oleic acid-coated nanoparticles into the solid-supported or the free-standing BLMs resulted in an increase of the Young’s modulus of elasticity measured by the nanoidentation and electrostriction methods [32]. This modulus is a measure of stiffness of the lipid bilayer and its increased value suggests that the membrane is prone to mechanical rupture in an alternating magnetic field by the vibrating superparamagnetic nanoparticles localized inside the bilayer walls. Even though the hydrophobic nanoparticles used (ca. 7 nm in diameter) seemed to be too large to be hosted fully in the lipid bilayer, which is 3–4 nm thick, the same explanation can be proposed here [48,49]. Earlier, the rupture of magnetoliposomes was confirmed by TEM and the doxorubicin outflow from magnetoliposomes was also shown under LF-AMF of 50 Hz frequency and 10 mT magnetic field intensity [32]. Therefore, we used this approach to induce the methotrexate release from magnetocubosomes, assuming that the mechanical vibration of magnetic nanoparticles rather than the localized heating of the MNPs’ vicinity would lead to similar local disruption of magnetocubosomes and MTX release under LF-AMF. A freshly prepared sample of 0.5 mg/mL magnetocubosome dispersion was placed inside the inductor coil. After a specified time, the absorbance was measured at 303 nm (Figure 8). The application of AMF led to an increase in the rate of drug release from the cubosomes. The relatively large data scatter, regardless of the number of repetitions, was ascribed to the ex situ approach, where the sample was transferred from the inductor coil to the spectrophotometer chamber. Nevertheless, two important facts can be extrapolated from our results. First, the release takes place very fast and reaches saturation within the first 5 min of the application of AMF. This release appears to be much faster than in the case of magnetoliposomes [32]. After this time, the amount of drug released from the magnetocubosomes reached 2.6 μg/mg (30% of the drug released when compared to the initial drug load). Second, over the same time period, the release from magnetocubosomes without AMF stimulus was only ca. 0.16 μg/mg, hence 2% of the drug released when compared to the initial drug load. The dashed curve shown in Figure 8, even though obtained by fitting the results to the Korsmeyer‒Peppas model for magnetocubosomes stimulated with AMF, is the possible outcome. Nevertheless, it is clear that the application of LF-AMF triggers a larger and faster release of the drug from the magnetocubosomes. Furthermore, since the frequency of the AMF stimulus and the relaxation time of the magnetic nanoparticles used were insufficient to trigger the magnetothermal effect (vide supra and reference [32]), we presumed that the alternating magnetic field at a low frequency of 50 Hz caused mechanical vibration of the magnetic nanoparticles that can rupture to some extent the internal structure of magnetocubosomes, facilitating the drug’s delivery from the aqueous channels of the cubosomes. Larger disturbances may lead to changes in the internal structure and possible phase transition [45].

## 4. Conclusions

Methotrexate is a widely used drug; however, due to its toxic side effects, it should be delivered to organisms encapsulated using an appropriate drug delivery vehicle to prevent healthy cells from being exposed to its toxic influence. We have shown that the lipidic liquid crystalline cubic phase can be a promising matrix for such a task. The release profiles of the drug from the films of the cubic phases placed at electrodes are described by the Higuchi model. The removal of the drug from the cubic phase was facilitated by an increase in temperature. The cubic phase dispersed into cubosomes should be even more attractive as MTX nanocarriers since they retain the internal structure of the parent bulk phase while the viscosity of the system is reduced, which makes it easier to apply.

Hydrophobic magnetic nanoparticles could be easily incorporated into the cubic phase nanoparticles. Since cubosomes are made of lipids organized in very expanded, curved bilayers they should be prone to the mechanical vibration of numerous magnetic nanoparticles present in these bilayers. Joniec et al. observed an increase in the Young’s modulus due to the presence of hydrophobic magnetic nanoparticles in magnetoliposomes loaded with doxorubicin [32]. The rupture of magnetoliposomes placed in the low-frequency alternating magnetic field due to mechanical vibration was observed by TEM and the release of doxorubicin from magnetoliposomes in the LF-AMF of 50 Hz frequency and 10 mT magnetic field intensity was monitored with fluorescence and voltammetry. Methotrexate release from the magnetocubosomes placed in the LF-AMF of the same frequency and field intensity lead also to local disruption of magnetocubosomes and release of MTX, as confirmed with UV-Vis spectroscopy. After the same length of time, MTX was barely released from magnetocubosomes in the absence of LF-AMF, which confirmed the role of magnetic field in triggering drug release from the cubosome.

These results show that magnetocubosomes are interesting hybrid nanostructures that may accommodate a suitable amount of methotrexate, due to the structure—larger than other carriers. The rate and efficiency of methotrexate release at the appropriate sites may be controlled, e.g., by applying an alternating magnetic field. Magnetic field can be used to relocate the dispersion of magnetocubosomes without any leakage of the magnetic nanoparticles from the cubosome lipid phase to the solution. The new hybrid dispersed nanomaterial is certainly promising as a carrier and requires in vitro and in vivo studies. Leesajakul et al. [50] studied the stability of cubosomes with Pluronic 127 as the stabilizer in plasma and reported that HDL, LDL, and albumin interacted with cubosome particles. The destabilization of cubosomes in plasma resulted in smaller particle remnants These authors suggested that long circulation time of an incorporated substance in cubosomes was due to the sustained behavior of cubosomes’ remnant particles. In a recent paper, Tran et al. [51] confirmed the accumulation of both cubosomes and hexosomes in the livers and spleens of mice up to 20 h post-injection. Their biodistribution and histology analysis results indicated that cubosomes and hexosomes should be considered as candidates for macrophage-associated theranostic systems. The interactions of cubosomes with proteins were also studied by, e.g., Falchi et al. [52] and Biffi et al. [53]. These authors show that the stability of cubosomes in the body fluids depends on the lipid and especially polymer used in the in vivo studies. Pluronic F108 and PEG were found to be the best stabilizers of cubosomes and hexosomes in the organism. We presented in vitro cytotoxicity studies for monoolein cubosomes and hexosomes containing other drugs [54,55,56]), confirming the stability of these nanoparticles in the cell environment. Further in-depth studies are needed to prove their utility for medical applications.

## Figures and Tables

**Table 1:** Results of SAXS measurements for the monoolein cubic phase with and without the drug: phase symmetry, lattice parameter *a*, lipid length *l*, and water channel diameter *d_w_*.

	T [°C]	Symmetry	a [nm]	l [nm]	d_w_ [nm]
MO/aq* **60/40%**	25	*Pn3̅m*	10.2	1.7	4.5
	37	*Pn3̅m*	9.5	1.6	4.2
MO/MTX/aq* **59/1/40%**	25	*Pn3̅m*	10.3	1.8	4.5
	37	*Pn3̅m*	9.8	1.7	4.3
MO/MTX/aq* **58/2/40%**	25	*Pn3̅m*	10.3	1.8	4.6
	37	*Pn3̅m*	9.7	1.6	4.3

*‘aq’ is used as an abbreviation for the phosphate buffer.

**Table 2:** Release characteristics of methotrexate from LCPs.

	Korsmeyer–Peppas			Higuchi	
% MTX	*n*	*R* ^2^	k [%/h^n^]	k_H_ [%/h]	*R* ^2^
1.0 at 25 °C	0.44 ± 0.05	0.986 ± 0.004	56.08 ± 5.40	47.49 ± 1.49	0.977 ± 0.010
1.0 at 25 °C CUV-Vis	0.44 ± 0.01	0.998 ± 0.001	61.20±1.92	58.78 ± 1.32	0.997±0.001
1.0 at 37 °C	0.65 ± 0.19	0.921 ± 0.102	66.36 ± 6.32	a	a
0.5 at 25 °C	0.41 ± 0.03	0.967 ± 0.006	62.34 ± 2.22	40.51 ± 9.49	0.943 ± 0.038
0.5 at 37 °C	0.56 ± 0.11	0.950 ± 0.043	61.38 ± 8.76	66.22 ± 4.21	0.966 ± 0.020
0.5 and 2% MNPs at 25 °C	0.47 ± 0.06	0.949 ± 0.014	60.60 ± 2.73	62.68 ± 5.85	0.945 ± 0.021
0.25 at 25 °C	0.45 ± 0.08	0.975 ± 0.005	57.12 ± 3.80	53.97 ± 3.38	0.982 ± 0.008
0.25 at 37 °C	0.57 ± 0.09	0.943 ± 0.014	63.51 ± 4.18	72.21 ± 4.11	0.962 ± 0.009

a: not determined.

## Supplementary Materials

The following are available online at https://www.mdpi.com/2079-4991/9/4/636/s1, Figure S1: Scheme of chemical structure of methotrexate (MTX) and folic acid (FA). S2: Equations used for the determination of Symmetry, lattice parameter, lipid length and diameter of water channels of the cubic phases. Figure S3: Scheme of the reduction process of methotrexate. Figure S4: Release profiles of MTX from a cubic phase in pH 7.4 at 25 and 37 °C. Figure S5: DPV on GC electrode modified with phases without and with magnetic nanoparticles and the release profiles of MTX from LCPs at pH 7.4 at 25 °C. Figure S6: The size and zeta potential of magnetocubosomes containing MTX determined with DLS at 25 °C. Figure S7: Standard calibration curve for methotrexate based on measurement at 303 nm in 0.1 M phosphate buffer, pH 7.4. Figure S8: Electron cryo-microscopy images of cubosomes and magnetocubosome. Video S9: Movement of magnetocubosomes in magnetic field.

## Abbreviations

MTX	methotrexate
SAXS	small angle X-ray scattering
LCP	liquid crystalline phase
DDS	drug delivery system
AMF	alternating magnetic field
MO	monoolein
MNPs	magnetic nanoparticles
DLS	dynamic light scattering
Cryo-TEM	cryogenic transmission electron microscopy
DPV	differential pulse voltammetry
GC	glassy carbon
GCE	glassy carbon electrode
SWV	square-wave voltammetry
CV	cyclic voltammetry
EE	entrapment efficiency
