# Supplementary material for: Lipidic Liquid Crystalline Cubic Phases and Magnetocubosomes as Methotrexate Carriers
Source: Nanomaterials (Basel). 2019 Apr 19;9(4):636. doi: 10.3390/nano9040636 (PMC6524136; doi:10.3390/nano9040636)
Supplement: Supplementary file 1 [file nanomaterials-09-00636-s001.pdf]

Supporting Information

Lipidic Liquid Crystalline Cubic Phases and Magnetocubosomes as Methotrexate Carriers

Monika Mierzwa, Adrianna Cytryniak, Paweł Krysiński and Renata Bilewicz *

Faculty of Chemistry, University of Warsaw, Pasteura 1, PL 02-093 Warsaw, Poland
* Corresponding author: bilewicz@chem.uw.edu.pl

S1

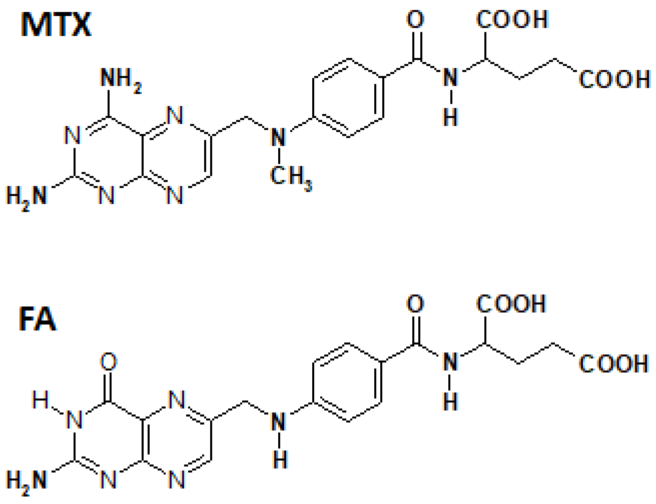

Scheme of chemical structure of methotrexate (MTX) and folic acid (FA).

S2

The SAXS measurement, allows to obtain one dimensional function of scattering intensities in function of q - I(q) where q (nm^{-1}) is the length of the scattering vector.

The scattering vector is related to the scattering angle—θ—and the wavelength of radiation—λ (in our case it is $\lambda_{Cu,K\alpha}$ = 0.1542 nm) by the relation [1]:

$$q = \frac{4\pi sin\theta}{\lambda} \qquad (1)$$

The q_i peak values are marked starting with q_0 and the ratio q_i/q_0 is calculated. The ratio defines the phase symmetry and Miller indices of the Bragg peak. The lattice parameter *a* (nm^{-1}) is calculated from the distance between 2 reflection plans *d*.

For cubic phases:

$$a = d_{hkl}\sqrt{h^2+k^2+l^2} \qquad (2)$$

$$d_{hkl} = \frac{2\pi}{q} \qquad (3)$$

$$a = \frac{2\pi}{q}\sqrt{h^2+k^2+l^2} \qquad (4)$$

Size of the water channels was calculated using the lattice parameter and the composition of cubic phases:

$$\phi_w = \frac{Cw}{Cw+(1-Cw)\frac{\rho w}{\rho l}} \quad (5)$$

where ϕ_w: water volume fraction, C_w: water weight fraction, ρw: density of water = 0.997 g/cm^3, ρl:density of lipid, in our case ρMO = 0.942 g/cm^3.

Lipid volume fraction was determined from the equation:

$$\phi_l = 1 - \phi_w \quad (6)$$

Lipid chain length (l) was determined by solving the following equation [2]:

$$\varphi lipid = 2\delta\left(\frac{l}{a}\right) + \frac{4}{3}\pi\chi\left(\frac{l}{a}\right)^3 \quad (7)$$

δ: ratio of the minimal surface in a unit cell to the quantity (unit cell volume)$^{2/3}$, χ: Euler–Poincare′ characteristic, *a*: lattice parameter of corresponding phase, *l*: lipid chain length/monolayer thickness.

Radius of water channels - r_w was obtained by equation [3]:

$$r_w = \left(\frac{-\delta}{2\pi\chi}\right)^{1/2} a - l \quad (8)$$

1. Kulkarni, Ch.V.; Wachter, W.; Iglesias-Salto, G.; Engelskirchenb, S.; Ahualliac, S. Monoolein: a magic lipid? *Phys. Chem. Chem. Phys.* **2011**, *13*, 3004–3021, doi:10.1039/c0cp01539c2.
2. Turner, D.C.; Wang, Z.-G.; Gruner, S.M.; Mannock, D.A.; McElhaney, R.N. Structural Study of the Inverted Cubic Phases of di- Dodecyl Alkyl-β-D-Glucopyranosyl-rac-Glycerol. *J. Phys.* **1992**, 2, 2039–2063, doi:10.1051/jp2:1992250.
3. Anderson, D.M.; Gruner, S.M.; Leibler, S. Geometrical Aspects of the Frustration in the Cubic Phases of Lyotropic Liquid Crystals. *Proc. Natl. Acad. Sci. USA* **1988**, *85*, 5364–5368.

S3

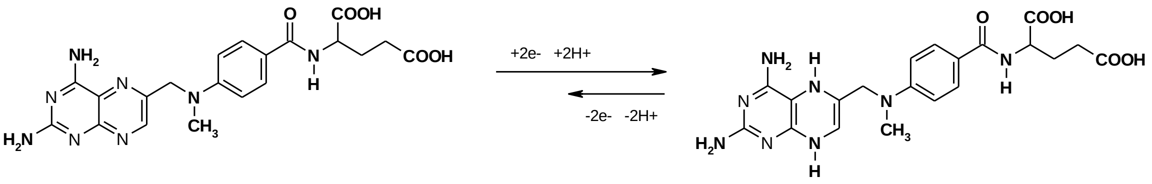

Scheme of the reduction process of methotrexate [1].

1. Pontinha, A.D.R.; Jorge, S.M.A.; Diculescu, V.C.; Vivan, M.; Oliveira-Brett, A.M. Antineoplasic Drug Methotrexate Redox Mechanism Using a Glassy Carbon Electrode. *Electroanalysis* **2012**, *24*, 917–923, doi:10.1002/elan.201100558.

S4

Release profiles of MTX from a cubic phase in pH 7.4 at 25 [A] and 37 °C [B].

S5

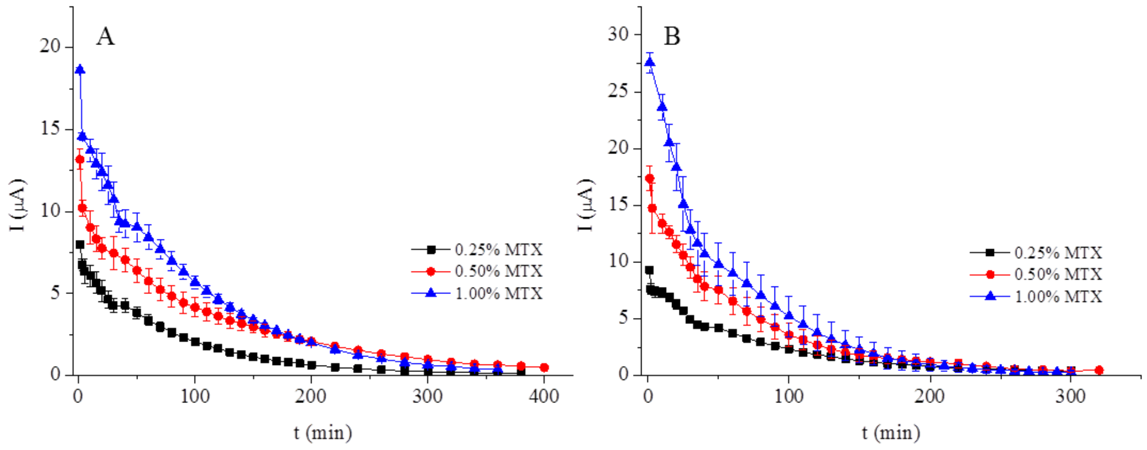

DPV on GC electrode modified with phases without [A] and with [B] magnetic nanoparticles and the release profiles of MTX from LCPs [C] at pH 7.4 at 25 °C.

S6

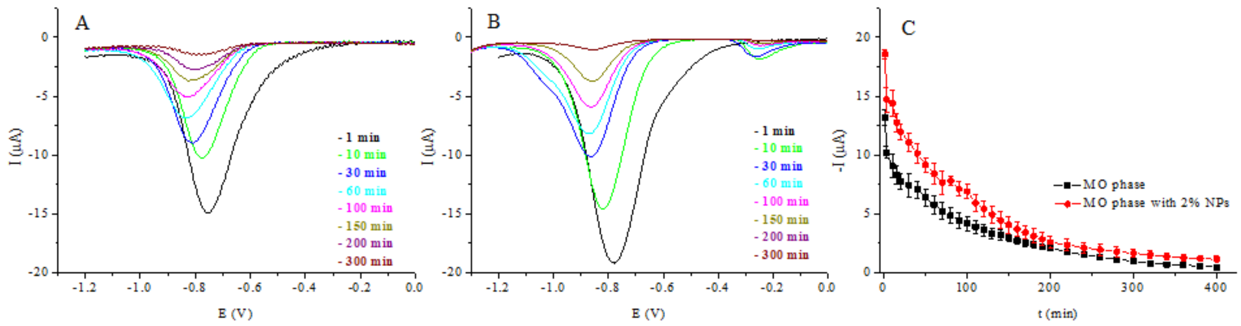

The size [A] and zeta potential [B] of magnetocubosomes containing MTX determined with DLS at 25 °C.

S7

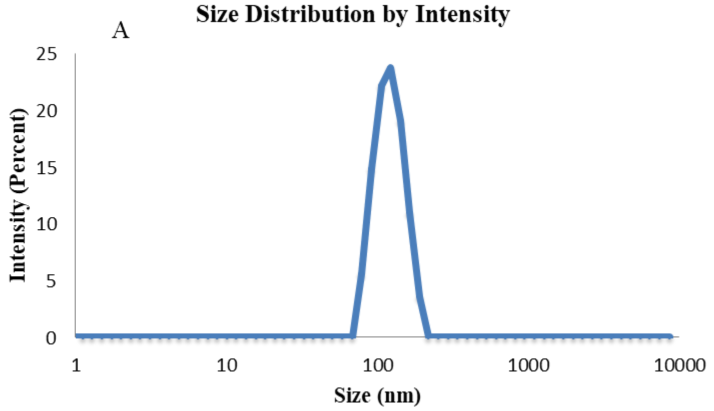

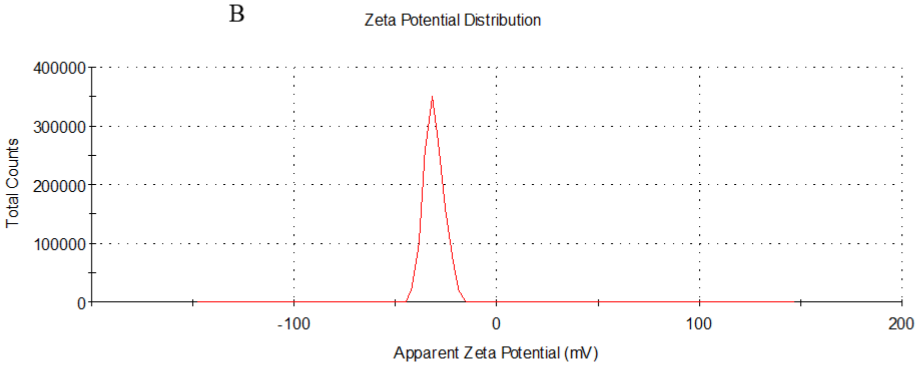

Standard calibration curve for methotrexate based on measurement at 303 nm in 0.1 M phosphate buffer, pH 7.4.

S8

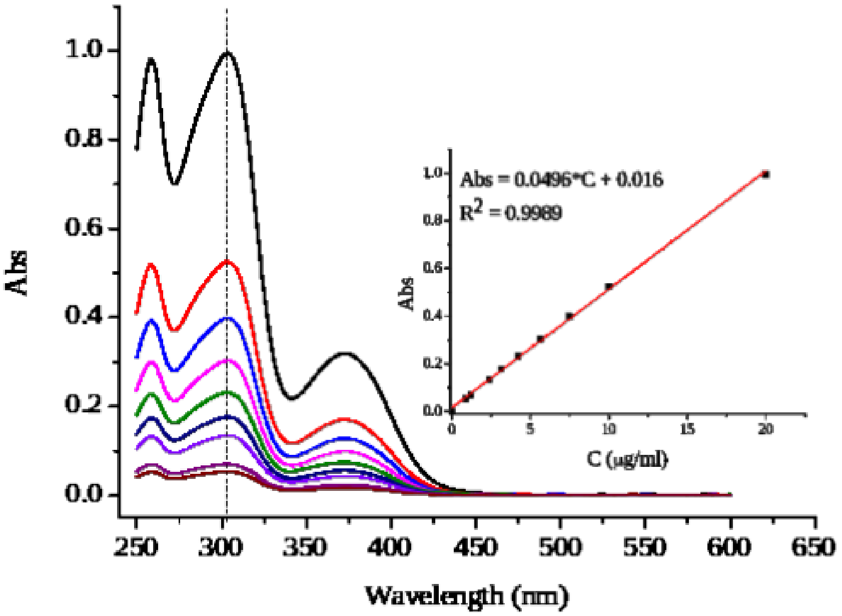

Electron cryo-microscopy images of cubosomes [A] and magnetocubosome [B].

S9

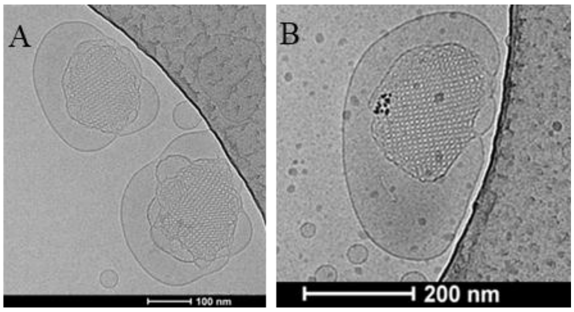

Movement of magnetocubosomes in magnetic field.

https://www.youtube.com/watch?v=dY5wi2V3GH4
